# Applying Retinal Vascular Structures Characteristics Coupling with Cortical Visual System in Alzheimer’s Disease Spectrum Patients

**DOI:** 10.3390/brainsci13020339

**Published:** 2023-02-16

**Authors:** Lianlian Wang, Zheqi Hu, Haifeng Chen, Xiaoning Sheng, Ruomeng Qin, Pengfei Shao, Zhiyuan Yang, Weina Yao, Hui Zhao, Yun Xu, Feng Bai

**Affiliations:** 1 Department of Neurology, Nanjing Drum Tower Hospital Clinical College of Jiangsu University, Nanjing 210008, China; 2 Department of Neurology, Nanjing Drum Tower Hospital of the Affiliated Hospital of Nanjing University Medical School, the State Key Laboratory of Pharmaceutical Biotechnology, Institute of Brain Science, Nanjing University, Nanjing 210008, China; 3 Jiangsu Province Stroke Center for Diagnosis and Therapy, Nanjing 210008, China; 4 Nanjing Neuropsychiatry Clinic Medical Center, Nanjing 210008, China; 5 Geriatric Medicine Center, Affiliated Taikang Xianlin Drum Tower Hospital, Medical School of Nanjing University, Nanjing 210008, China

**Keywords:** Alzheimer’s Disease, retinal vascular structures, cortical visual system, optical coherence tomography angiography, functional magnetic resonance imaging

## Abstract

Cortical visual system dysfunction is closely related to the progression of Alzheimer’s Disease (AD), while retinal vascular structures play an important role in the integrity of the function of the visual network and are a potential biomarker of AD. This study explored the association between the cortical visual system and retinal vascular structures in AD-spectrum patients, and it established a screening tool to detect preclinical AD based on these parameters identified in a retinal examination. A total of 42 subjects were enrolled and were distributed into two groups: 22 patients with cognitive impairment and 20 healthy controls. All participants underwent neuropsychological tests, optical coherence tomography angiography and resting-state fMRI imaging. Seed-based functional connectivity analysis was used to construct the cortical visual network. The association of functional connectivity of the cortical visual system and retinal vascular structures was further explored in these subjects. This study found that the cognitive impairment group displayed prominently decreased functional connectivity of the cortical visual system mainly involving the right inferior temporal gyrus, left supramarginal gyrus and right postcentral gyrus. Meanwhile, we observed that retinal vascular structure characteristics deteriorated with the decline in functional connectivity in the cortical visual system. Our study provided novel insights into the aberrant cortical visual system in patients with cognitive impairment that strongly emphasized the critical role of retinal vascular structure characteristics, which could be used as potential biomarkers for diagnosing and monitoring the progression of AD.

## 1. Introduction

Alzheimer’s Disease (AD) is the most prevalent cause of dementia, accounting for 60 to 70 percent of all cases globally. Currently, there are approximately 50 million individuals worldwide who are affected by dementia, and with increasing life expectancies, this number is projected to rise to 152 million by 2050 [1]. AD is characterized by the formation of plaques composed of amyloid proteins and tangles comprised of hyper-phosphorylated tau [2]. The Alzheimer’s Disease (AD) spectrum encompasses a range of conditions, including subjective cognitive decline (SCD), amnestic mild cognitive impairment (aMCI), non-amnesic mild cognitive impairment (naMCI), and confirmed AD. Mild cognitive impairment (MCI) is considered a transitional stage between the normal aging process and AD and serves as a crucial component of the AD spectrum [3]. Individuals with Alzheimer’s Disease (AD) typically exhibit multi-dimensional cognitive dysfunction [4], including visual impairments such as reduced visual acuity, decreased sensitivity to color and contrast, and visual field deficits, particularly in the early stages of the disease. These visual impairments may become clinically apparent among AD patients [5,6].

The hallmark feature of early Alzheimer’s Disease (AD) is memory impairment resulting from the degeneration of the nerve cells in the medial temporal lobe. This characteristic presents as a typical manifestation of the disease in its early stages [7,8]. As the progression of Alzheimer’s Disease (AD) advances, the neurodegenerative process gradually spreads from the medial temporal lobe to the temporal and parietal cortex, eventually affecting a significant portion of the cerebral cortex [7]. Additionally, it is common for individuals with Alzheimer’s Disease (AD) to exhibit cortical visual dysfunction [9]. The cortical visual system is widely accepted as being composed of two anatomically and functionally distinct pathways: the ventral occipitotemporal pathway, which is responsible for object perception, and the occipitoparietal pathway, which facilitates object localization and visual guidance [10] and is involved in visual processing. Several studies have revealed that changes in the retina may reflect the neuropathological changes in the brain associated with Alzheimer’s Disease (AD), particularly the atrophy of the occipital, parietal, and temporal lobes [11,12,13]. The occipital cortex has traditionally been considered the visual association area and is highly susceptible to the deposition of amyloid plaques and neurofibrillary tangles, leading to a range of visual impairments. The atrophy in the occipital cortex has been recognized as an early and prominent aspect of neurodegeneration in Alzheimer’s Disease (AD) [1,14].

The retina, which is an extension of the central nervous system, shares a common embryonic origin with the brain regions responsible for cognition [1,15]. Previous studies have reported macular thickness reduction [16], ganglion cell-inner plexiform layer (GC-IPL) thickness [17] and retinal nerve fiber layer(RNFL) thinning [15,18], and retinal vascular density and ocular perfusion reduction [19] in AD patients as well as in Parkinson’s disease and multiple sclerosis [20,21]. It is noteworthy that the retinal vascular system can be utilized as a window for evaluating the impact of vascular pathology on Alzheimer’s Disease (AD) [22]. Our hypothesis is that alterations in the retinal microvasculature may reflect changes in brain function. To test this hypothesis, the present study aimed to explore the relationship between the cortical visual system and retinal vascular structures in patients with the Alzheimer’s Disease (AD) spectrum. Additionally, the study aimed to establish a screening tool for the early detection of preclinical AD using parameters derived from retinal examinations.

## 2. Materials and Methods

### 2.1. Participants

**Figure 1:**
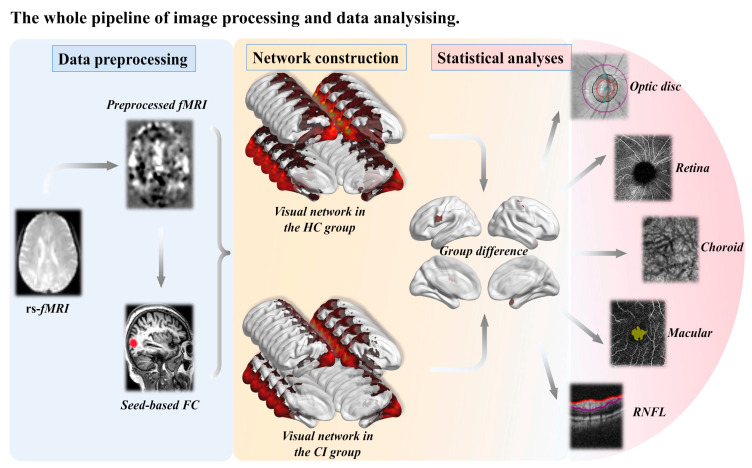
A flowchart illustrates the entire process of image processing and data analysis, which involves the subjects of cognitive impairment (CI) and healthy controls (HC), and measures retinal nerve fiber layer (RNFL).

This cross-sectional study was approved by the Ethics Committee of Nanjing Drum Tower Hospital and informed consent was obtained from 22 individuals with cognitive impairment (CI) and 20 healthy control (HC) participants. The criteria for inclusion of CI subjects were as follows [23]: (a) A report of a perceived change in cognition by the participant, an informant, or a skilled clinician. (b) Evidence of impairment in one or more cognitive domains (memory, attention, executive function, language, visuospatial skills) beyond what would be expected for the individual’s age and education level. (c) Retention of independence in functional abilities, meaning that the patients may have mild difficulties in performing complex tasks, but should be capable of maintaining their independence with minimal support. (d) Absence of severe dementia. The study recruited 20 healthy control (HC) subjects with an age range of 50–80 years and with an intraocular pressure of 10–21 mmHg, who underwent a slit lamp examination to exclude retinopathy. Inclusion criteria for HC subjects were: (a) No history of type 1 or 2 diabetes. (b) No history of glaucoma, age-related macular degeneration, retinal vein obstruction, uveitis, or other ophthalmic diseases. (c) No history of severe cardiac insufficiency, malignant tumors, acute infectious diseases, pregnancy, or autoimmune diseases. (d) No history of trauma or surgery in the past 3 months. (e) No history of alcohol addiction, drug abuse, or other diseases that could lead to cognitive decline. (f) No history of severe anxiety or depression, mental illness, or congenital hypoplasia. (g) No contraindications to magnetic resonance imaging (MRI). (h) No severe vision, hearing, language, or physical activity disorders. (i) Ability to tolerate functional MRI. (j) No magnetic resonance findings of cerebral infarction, cerebral hemorrhage, or brain tumors. (k) No severe depression, anxiety, or other effects that could impact the test. (l) Ability to tolerate optical coherence tomography angiography (OCTA). The study pipeline is depicted in Figure 1.

### 2.2. Neuropsychological Assessments

All participants in the study underwent a standardized neuropsychological evaluation protocol conducted by an experienced neuropsychologist. The evaluation consisted of a general cognitive examination and multiple cognitive domain assessments. General cognitive function was evaluated using the Mini-Mental State Examination (MMSE) and the Beijing version of the Montreal Cognitive Assessment (MoCA-BJ). The study used the MoCA-BJ to detect healthy controls (HC) and cognitive impairment (CI). The optimal cutoff points for MoCA-BJ were determined based on education level, with the cutoff for subjects with 7 or more years of education being 24/25, 19/20 for subjects with 1–6 years of education, and 13/14 for subjects with no formal education. Raw examination scores were transformed into Z-scores to calculate each cognitive domain performance. Episodic memory was calculated as the mean of the Z-scores of the Auditory Verbal Learning Test-delayed recall and the Wechsler Memory Scale Visual Reproduction-delayed recall. The visuospatial function was calculated as the mean of the Z-scores of the Clock Drawing Test and Visual Reproduction-copy. Information processing speed was calculated as the average Z-scores of the Trail Making Test-A, Stroop Color and Word Tests A and B. Executive function was calculated as the average Z-scores of the TMT-B and Stroop C.

### 2.3. Optical Coherence Tomography Imagine

The subjects underwent imaging by an experienced ophthalmologist using a spectral-domain optical coherence tomography angiography (OCTA) machine, specifically the Cirrus HD-5000 AngioPlex machine manufactured by Carl Zeiss Meditec. This machine was capable of scanning at a speed of 68,000 A-scans per second. Two image sizes were captured, 3 mm × 3 mm and 6 mm × 6 mm, both of which were centered on the fovea. Images that were of poor quality with a signal strength below 7/10, or with segmentation errors, low resolution, motion artifacts, or projection artifacts, were excluded from the analysis. The full-layer retinal scans of the superficial capillary plexus (SCP) were segmented using OCTA software (Version 10.0, 2016, Carl Zeiss Meditec), which employed the Early Treatment Diabetic Retinopathy Study (ETDRS) grid overlay to quantitatively determine the mean vascular density (VD) and perfusion density (PD) of the SCP images in the central macula, both in the 3 mm × 3 mm and 6 mm × 6 mm sizes. The total length of the perfused vasculature system per unit area was calculated as the mean vascular density (VD) and was expressed in units of inverse millimeters. The total area of the perfused vasculature system per unit area in the region of measurement was determined as the perfusion density (PD). The VD and PD were automatically calculated for the 3-mm circle and 3-mm ring regions of the 3 mm × 3 mm optical coherence tomography angiography (OCTA) images and for the 6-mm circle, 6-mm ring, and 3-mm ring regions of the 6 mm × 6 mm OCTA images. The fovea avascular zone (FAZ) was automatically segmented and quantified using the OCTA software (Version 10.0; 2016; Carl Zeiss Meditec), which uses the Early Treatment Diabetic Retinopathy Study (ETDRS) grid overlay. The accuracy of the automatic segmentation of the FAZ region was verified through manual examination. In addition to the retinal scans, 512 × 128 macular cube and 200 × 200 optic disc cube scans were also obtained, and the mean RNFL thickness was determined using a 3.46-mm diameter circle centered on the optic disc.

### 2.4. Imaging Analysis

#### 2.4.1. Neuroimaging Data Acquisition

All subjects were scanned using a Philips 3.0-T scanner with a homogeneous birdcage head coil to minimize head movements. Before scanning, participants were instructed to keep their eyes closed, refrain from thinking and minimize movements during data acquisition. The compliance with these instructions was confirmed through a questionnaire administered after the scan, which indicated that none of the subjects had fallen asleep during the procedure. High-resolution T1-weighted sagittal images obtained through turbine rapid echo acquisition covering the entire brain were as follows: repetition time (TR) = 9.8 ms, flip angle (FA) = 8°, echo time (TE) = 4.6 ms, acquisition matrix = 256 × 256, thickness = 1.0 mm, FOV = 250 × 250 mm^2^, number of slices = 192. The resting-state functional scans covering 230 volumes were obtained using a gradient-recalled echo planar imaging sequence: TR = 2000 ms, FA = 90°, TE = 30 ms, acquisition matrix = 64 × 64, thickness = 4.0 mm, number of slices = 35, FOV = 240 × 240 mm^2^.

#### 2.4.2. Image Preprocessing

Analysis of brain imaging data was conducted with (DPABI 2.3, http://rfmri.org/DPABI (accessed on 21 November 2022)) software [24], which is based on statistical parametric mapping (SPM12, http://www.fifil.ion.ucl.ac.uk/spm (accessed on 21 November 2022)). The initial ten volumes of each participant were removed to allow for adjustment to the scanning noise and achieve signal equilibrium. The subsequent 220 volumes were then subjected to correction for the temporal difference in acquisition between slices and were realigned to the initial volume. The head motion parameters were then calculated, and it was established that no participant exhibited head movements that exceeded 3 mm in any of the x, y or z directions or rotation exceeding 3°. The resulting functional data underwent spatial normalization into the Montreal Neurological Institute (MNI) space using the mean EPI image as the source volume. The data were then resampled into 3 × 3 × 3 mm^3^ voxels and smoothed with a 6 × 6 × 6 mm Gaussian kernel. The removal of nuisance covariates, such as Friston-24 head motion parameters and the signals from white matter and cerebrospinal fluid, was performed through regression analysis. Subsequently, temporal filtering in the range of 0.01–0.08 Hz was applied to the time series data. These procedures were conducted on the entire brain using regression analysis.

#### 2.4.3. Definition of Visual Network

Seed-based functional connectivity analysis was used to construct the resting-state cortical visual system. We selected 6 mm radius spheres centered at the right middle occipital gyrus (MNI space: 39 −88 10) [25], which served as the seed region. The mean time series of the right middle occipital gyrus was computed for each subject as the reference time course for the cortical visual system. Pearson’s cross-correlation analysis was applied to determine the relationship between the seed time course and the time course of all brain voxels, and Fisher’s z-transformation was applied to enhance the normality of the correlation coefficients. This resulted in the generation of a functional connectivity map for each subject. To build the cortical visual system, a one-sample *t*-test was performed to identify the brain regions that displayed a positive correlation with the right middle occipital gyrus in all subjects.

### 2.5. Statistics Analysis

The statistical analysis was conducted using SPSS 26.0 (SPSS Inc., Chicago, IL, USA), a statistical software commonly used in the social sciences. The Chi-squared (χ^2^) test and two-sample t-test were employed to compare demographic, neuropsychological, and retinal data and variables such as age and gender. A significance threshold of *p* < 0.05 was established for all statistical procedures. A two-tailed t-test was performed on maps of two groups using the DPABI Data Analysis Toolkit (DPABI, http://www.dpabifmri.net (accessed on 21 November 2022)) with years of education as covariates. The result was set at individual voxel *p* < 0.005, cluster size > 270 mm^3^. The average functional connectivity strength of each significant region of interest was extracted from each subgroup using REST 1.7. Finally, the average functional connectivity strength of each significant region was compared between the two groups. A spearman correlation analysis was performed to evaluate the association between the cortical visual system and retinal vascular structures.

## 3. Results

### 3.1. Demographic and Clinical Characteristics

Demographic and clinical information were provided in Table 1. The population comprised 24 women (57.1%) and 18 men (42.9%). There was no significant difference for age, gender distribution and VR-C between CI and HC groups (*p* > 0.05). One of the patients only conducted MMSE and MoCA because of the COVID-19 epidemic, and the average of the upper and lower patients was taken during the analysis to fill in. The CI group exhibited significantly lower scores in MMSE (*p* < 0.001), MoCA (*p* < 0.001), information processing speed (*p* < 0.001), episodic memory (*p* < 0.001), visuospatial processing function (*p* < 0.002), and executive function (*p* < 0.001) than the HC group.

### 3.2. Retinal Vascular Structures Data

In nine patients one of the eyes was excluded due to trauma, age-related macular and other ophthalmic diseases by slit lamp examination, retaining data on healthy eyes. The analysis of the data is the average of the two eyes with complete data, and the data with one eye are directly selected. As shown in Table 2, there was no significant difference for all retinal structural parameters under the statistically significant at the 0.05 level between the two groups. However, we could find a slight decrease in Intra-layer VD, Completed VD, Central PD, Intra-layer PD, Completed PD, Optic disc area, Mean C/D, Vertical C/D, Optic cup size, FAZ circularity and RNFL, and a slight increase in FAZ area, FAZ perimeter in the CI when compared to HC.

### 3.3. Cortical Visual System Reconstruction

**Figure 2:**
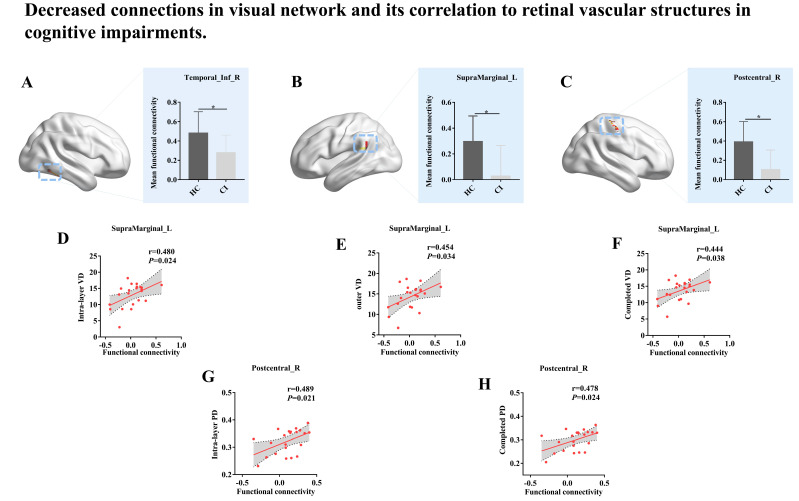
Decreased connections in visual network and its correlation to retinal vascular structures in cognitive impairments. (**A**−**C**) showed the abnormal changes in visual network in CI group: greater decreases in CI group compared with HC group, such as right inferior temporal gyrus, left supramarginal gyrus and right postcentral gyrus. The functional connectivity strength in left supramarginal gyrus was associated with Intra-layer VD (6 × 6 mm, (**D**)), Completed VD (6 × 6 mm, (**E**)) and Completed VD (6 × 6 mm, (**F**)), while the functional connectivity strength in right postcentral gyrus was related to Intra-layer PD (3 × 3 mm, (**G**)) and Completed PD (3 × 3 mm, (**H**)) in CI group. Statistically significant differences were defined as *p* < 0.05, indicated by the symbol *.

In line with the previous study, complex visual disturbances are described in AD-spectrum patients [26]. These disorders are visual processing disorders due to the disruption of corticocortical projections resulting from alterations in the cortical visual system [27]. The cortical visual system was associated with occipitoparietal and occipitotemporal lobes among the two groups (Figure 1). Compared to the HC group, the patients in the CI group displayed prominently decreased functional connectivity mainly involving the right inferior temporal gyrus (ITG), left supramarginal gyrus (SMG)and right postcentral gyrus (PCG) (Figure 2 and Table 3).

### 3.4. Associations of Retinal Vascular Structures Characteristics with Cortical Visual System

**Figure 3:**
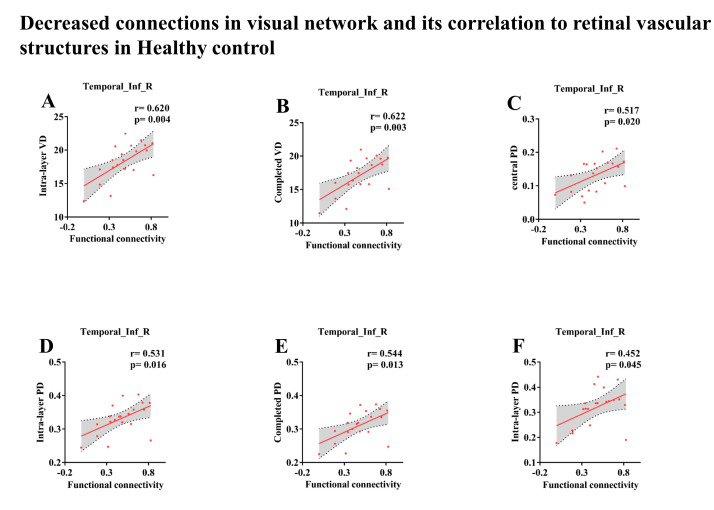
Decreased connections in visual network and its correlation to retinal vascular structures in Healthy control.The functional connectivity strength in right inferior temporal gyrus was associated with Intra-layer VD (3 × 3 mm, (**A**)), Completed VD (3 × 3 mm, (**B**)) and Central PD (3 × 3 mm, (**C**)) Completed PD (6 × 6 mm, (**D**)), Intra-layer PD (3 × 3 mm, (**E**)) and Intra-layer PD (3 × 3 mm, (**F**)) in HC group.

As shown in Figure 2, for subjects in the CI group, a significant correlation was found between the functional connectivity strength of the left SMG of the cortical visual system and Intra-layer VD (6 × 6 mm; r = 0.480, *p* = 0.024), Outer VD (6 × 6 mm; r = 0.454, *p* = 0.034), and Completed VD (6 × 6 mm, r = 0.444, *p* = 0.038), while the functional connectivity strength in the right PCG was a significantly positive correlation with Intra-layer PD (3 × 3 mm; r = 0.489, *p* = 0.021) and Completed PD (3 × 3 mm; r = 0.478, *p* = 0.024). Nonetheless, there was no significant effect of functional connectivity in the cortical visual system on FAZ area, FAZ perimeter, FAZ circularity, Optic disc area, Mean C/D, Vertical C/D, Optic cup size and RNFL. Besides, as demonstrated by the data presented in Table 4 and Figure 3, the subjects in the HC group displayed a significant positive correlation between the FC strength of Temporal_ Inf_ R in the VN and various measures of VD and PD. Specifically, a correlation was found between the FC strength and Intra-layer VD (3 × 3 mm; r = 0.620; *p* = 0.004, Figure 3A), Completed VD (3 × 3 mm; r = 0.622, *p* = 0.003, Figure 3B), Central PD (3 × 3 mm; r = 0.517, *p* = 0.020, Figure 3C), Intra-layer PD (3 × 3 mm; r = 0.531, *p* = 0.016, Figure 3D), Completed PD (3 × 3 mm; r = 0.544, *p* = 0.013, Figure 3E), and Intra-layer PD (6 × 6 mm; r = 0.452, *p* = 0.045, Figure 3F). No correlation was found between these indicators and age. We found no relationship between the above visual indicators and age, which correlated with different brain regions.

## 4. Discussion

The present study investigated the functional connectivity strength of the cortical visual system and its relationship to structural changes of the retina in AD-spectrum patients. We demonstrated that in addition to the decline in visuospatial processing function in subjects with cognitive impairment, it is also accompanied by a decrease in the functional connectivity strength of the cortical visual system. Our study further found a significant correlation between the functional connectivity strength of the cortical visual system and structural changes in the retina of AD-spectrum patients. We also found a significant correlation between the strength of functional connections of the cortical visual system and structural changes in the retina in the HC.

### 4.1. Underlying Mechanisms on Cognitive Impairment of Aberrant Cortical Visual System

Our findings highlight the importance of the occipital region in the development of cognitive impairment. This region is critical for high-level visual functions, including visual memory, integration of visual and tactile information, and object recognition. Visual impairment is often one of the first symptoms of AD and can have a profound impact on everyday activities [28]. 

The onset of visual impairment has been reported as an early manifestation in patients with Alzheimer’s Disease (AD), even prior to a definitive diagnosis [29]. The occipital region plays a crucial role in coordinating high-level visual functions such as visual memory, integration of visual and tactile information, and object recognition [30,31,32,33,34]. These findings align with previous studies that have indicated disruptions in visual pathways originating from the occipital lobes as seed points, which have been linked to the progression from normal cognition to cognitive impairment. However, the cause of these changes remains unclear, and it is yet to be determined whether they result from the disruption of large-scale neuronal networks or the local deposition of pathological agents [34,35]. Notably, individuals with reduced functional connectivity strength in the ITG, SMG, and PCG were found to have a higher likelihood of developing cognitive impairment. Previous research has established memory impairment as a hallmark of early Alzheimer’s Disease (AD) due to degeneration in the medial temporal lobe. The inferior temporal gyrus (ITG) has been found to have crucial neural connections with the structures in the medial temporal lobe cortex and has been implicated in the prodromal phase of the disease as a potential basis for early AD-related clinical dysfunction [36]. Neuroimaging studies have indicated the crucial role of the inferior parietal lobule (IPL), specifically the supramarginal gyrus (SMG), in visual word recognition [37]. Consequently, damage to the SMG or visual pathways connecting the occipital cortex and SMG may disrupt memory for visual words [38,39]. Additionally, the precentral gyrus (PCG) has been identified as responsible for the systematic integration of bilateral body parts, including somatic and visual information [40]. Our study investigated the relationship between the PCG and changes in retinal microvascular diseases. Further investigation is necessary to determine the role of the visual network associated with the occipital lobe in the progression from healthy control to cognitive impairment.

### 4.2. Retinal Markers Might Be Used as Potential Biomarkers for Diagnosing and Monitoring the Progression of AD

An increasing body of research has established that cortical visual dysfunction is a common occurrence in Alzheimer’s Disease (AD), often concurrently with other cognitive changes or, in some cases, preceding them [9,41,42]. Traditional diagnoses of AD have primarily relied on structural and functional imaging techniques such as magnetic resonance imaging (MRI) and positron emission tomography (PET), as well as invasive biomarkers such as cerebrospinal fluid (CSF) and blood measurements [43,44]. In comparison, eye and vision examinations for AD patients offer a less invasive and cost-effective alternative to current technology. The present study found that a decrease in the functional connection value of the visual network was associated with a reduction in the perfusion density and perfusion density integrity of the retinal vessels, indicating abnormalities in retinal microvascularization. The retina as a biomarker for neurodegenerative diseases has not been extensively explored, but previous research has established a correlation between retinal loss and neurocognitive decline [11,45]. For instance, optical coherence tomography angiography (OCTA) has been used to detect neurodegenerative vascular changes through decreased retinal vascular density and perfusion density in Alzheimer’s disease (AD) and mild cognitive impairment (MCI), as well as potentially preclinical AD [17,46,47,48]. Changes in the retinal microvascular system may reflect changes in the cerebral microvascular system in AD and MCI due to their anatomical, embryological, and physiological similarities [13]. Our study found that as the functional connection strength decreased in AD-spectrum patients, the retinal vascular density and perfusion density also decreased in the lingual sulcus middle gyrus (LSMG) and posterior cingulate gyrus (PCG) of the cognitively impaired (CI) group, suggesting that vascular dysfunction plays a crucial role in the pathogenesis of CI and that changes in cerebral perfusion exist before the onset of clinical symptoms [19,49]. A study on Diffusion Tensor Imaging (DTI) variables revealed a significant correlation between the retinal nerve fiber layer (RNFL) and visual pathways [15]. However, another examination of the occipital lobe, which encompasses the visual cortex, did not reveal a clear association with RNFL [12]. This discrepancy may stem from the small sample size studied, or the brain region associated with RNFL may not be located within the occipital lobe. In line with previous studies [17], our research found that functional connectivity (FC) in the visual network (VN) did not significantly affect the parameters of the foveal avascular zone (FAZ) area, circumference, roundness, optic disc area, average cup-to-disc ratio (C/D), vertical C/D, optical cup size. This result may be a consequence of the limited sample size in our study. Numerous studies have established the significant role of vascular factors in the pathogenesis of Alzheimer’s disease (AD) [50]. Previous research has also demonstrated that optical coherence tomography angiography (OCTA) can predict microcirculation changes associated with AD pathologically, via retinal biomarkers, as a rapid and non-invasive imaging technique [46,47,51]. Thus, retinal markers hold the potential to serve as promising biomarkers for diagnosing and monitoring the progression of AD in the near future. 

### 4.3. Strengths and Limitations

The participants in this study underwent comprehensive eye examinations, including slit lamp examinations, intraocular pressure measurements, and fundus examinations, effectively eliminating the potential impact of ocular diseases on the results. Additionally, we report, for the first time, the correlation between retinal microvascular parameters obtained via optical coherence tomography angiography (OCTA) and the functional connectivity of the visual network in individuals with cognitive impairments. However, several limitations should be noted. Firstly, this is a cross-sectional study, thus, caution should be exercised when interpreting the results and drawing conclusions about the effect of flow density on disease progression. Further research utilizing a longitudinal design and long-term follow-up of participants is needed. Secondly, after adjusting for age, sex, years of education, and other confounding variables, the association was not significant in multivariable linear regression. This result may be partially due to the limited sample size, and replication of these findings in larger patient populations is necessary for validation.

In conclusion, the results of this study demonstrate that individuals with cognitive impairment exhibit a significant reduction in functional connectivity within the cortical visual system, particularly in the right inferior temporal gyrus, left supramarginal gyrus, and right postcentral gyrus. Concurrently, our findings indicate that the deterioration of retinal vascular structure characteristics is positively correlated with declining functional connectivity in the cortical visual system. This study offers novel insights into the aberrant cortical visual system in patients with cognitive impairment, underscoring the crucial role of retinal vascular structure characteristics as potential biomarkers for the diagnosis and monitoring of Alzheimer’s disease (AD). Future research endeavors will focus on conducting longitudinal follow-up studies with larger sample sizes.

## Figures and Tables

**Table 1:** Demographics and clinical characteristics of HC and CI subjects.

Characteristic	HC	CI	*p* Value
	(*n* = 20)	(*n* = 22)	
Demogrraphics			
Gender(female/male)	11/9	7/15	0.129
Age, years	62.1 ± 7.56	66.91 ± 8.29	0.057
Education, years	13.65 ± 2.13	10.36 ± 2.57	<0.001 *
General cognitive			
MMSE	28.85 ± 1.18	25.41 ± 3.96	0.001 *
MoCA-BJ	26.90 ± 1.52	19.50 ± 4.35	<0.001 *
Composition scores of each cognitive domain			
Episodic memory	1.00 ± 1.63	−0.93 ± 1.42	<0.001 *
AVLT-DR	5.75 ± 2.81	3.16 ± 2.44	0.003 *
VR-DR	8.10 ± 4.22	3.39 ± 3.75	<0.001 *
Visuospatial processing function	4.15 ± 0.50	2.59 ± 2.04	0.002 *
CDT	3.85 ± 0.49	2.86 ± 1.32	0.005 *
VR-C	13.85 ± 0.49	12.05 ± 4.15	0.055
Information processing speed	1.50 ± 1.84	−1.37 ± 2.44	<0.001 *
TMT-A (inverse)	0.02 ± 0.01	0.01 ± 0.01	0.002 *
Stroop A (inverse)	0.07 ± 0.02	0.05 ± 0.02	0.001 *
Stroop B (inverse)	0.06 ± 0.02	0.04 ± 0.02	0.001 *
Executive function	0.98 ± 1.37	−0.89 ± 1.47	<0.001 *
TMTB (inverse)	0.012 ± 0.005	0.007 ± 0.004	0.001 *
Stroop C(inverse)	0.036 ± 0.010	0.026 ± 0.01	0.003 *

Values are reported as the mean ± standard deviation or as percentages. The gender effect was evaluated using a χ^2^ test, while the differences between groups were analyzed using a two-sample *t*-test. Statistically significant differences were defined as *p* < 0.05, indicated by the symbol *. The following acronyms are used throughout the text: HC, health control; CI, cognitive impairment; MMSE, mini mental state examination; MoCA, Montreal Cognitive Assessment; AVLT-DR, Auditory Verbal Learning Test-delayed recall; VR-DR, visual reproduction-delayed recall; CDT, Clock Drawing Test; VR-C, visual reproduction-copy; TMT-A and TMT-B, Trail Making Test-A and B; Stroop A, B, and C, Stroop Color and Word Tests A, B, and C.

**Table 2:** Structural changes of the retinal vascular structures between HC and CI subjects.

Characteristic	HC (*n* = 20)	CI (*n* = 22)	* *p* Value
Optic disc area	2.058 ± 0.542	1.956 ± 0.375	0.484
Mean C/D	0.568 ± 0.132	0.550 ± 0.112	0.647
Vertical C/D	0.512 ± 0.150	0.505 ± 0.108	0.86
Optic cup size	0.242 ± 0.218	0.194 ± 0.165	0.428
RNFL	94.475 ± 8.076	94.250 ± 10.689	0.599
Angiography 3 mm × 3 mm			
Intra-layer VD	18.300 ± 2.713	17.757 ± 2.671	0.518
Completed VD	17.090 ± 2.661	16.502 ± 2.510	0.467
Central PD	0.132 ± 0.047	0.122 ± 0.045	0.470
Intra-layer PD	0.332 ± 0.046	0.322 ± 0.0453	0.468
Completed PD	0.310 ± 0.046	0.308 ± 0.053	0.398
FAZ area	0.267 ± 0.083	0.307 ± 0.246	0.555
FAZ perimeter	2.296 ± 0.319	2.332 ± 0.583	0.804
FAZ circularity	0.625 ± 0.108	0.617 ± 0.224	0.883
Angiography 6 mm × 6 mm			
FAZ area	0.216 ± 0.082	0.246 ± 0.116	0.338
FAZ perimeter	1.984 ± 0.470	2.276 ± 0.733	0.136
FAZ circularity	0.672 ± 0.086	0.614 ± 0.123	0.087
Intra-layer VD	13.563 ± 2.923	12.911 ± 3.604	0.522
Outer VD	15.100 ± 2.261	14.323 ± 3.032	0.349
Completed VD	14.448 ± 2.305	13.646 ± 3.068	0.342
Central PD	0.102 ± 0.055	0.095 ± 0.062	0.723
Intra-layer PD	0.320 ± 0.075	0.307 ± 0.081	0.594
Outer PD	0.369 ± 0.060	0.357 ± 0.069	0.545
Completed PD	0.350 ± 0.060	0.338 ± 0.069	0.573

C/D, ratio of cup size to disc area; RNFL, retinal nerve fiber layer; FAZ, fovea avascular zone; VD, vascular density; PD, perfusion density. Indicates a statistical difference between groups, *p* < 0.05,indicated by the symbol *.

**Table 3:** Different brain regions in visual network between HC and CI subjects.

Regions	Cluster Size (mm^3^)	BA	Peak F Value	Peak MNI Coordinate
				x, y, z (mm)
Right ITG	324	37	3.6261	51 −51 −18
Left SMG	540	40	3.867	−57 −42 27
Right PCG	1161	3	3.8698	45 −27 66

The individual voxel *p* < 0.005 and cluster size > 270 mm^3^ were taken as meaning that there was a significant difference between groups. BA, Brodmann area; MNI, Montreal Neurological Institute; ITG, inferior temporal gyrus; SMG, supramarginal gyrus; PCG, postcentral gyrus; cluster size is in mm^3^.

**Table 4:** Correlative analysis between FC changes and structural changes of the fundus of HC and CI subject.

Brain Regions	Visual Data	r-Value	*p*-Value	Effects of FC Changes on Vision
Temporal_Inf_R				
	Intra-layer VD (3 mm × 3 mm)	0.62	0.004	Harmful
	Completed VD (3 mm × 3 mm)	0.622	0.003	Harmful
	Central PD (3 mm × 3 mm)	0.517	0.02	Harmful
	Intra-layer PD (3 mm × 3 m)	0.531	0.016	Harmful
	Completed PD (3 mm × 3 mm)	0.544	0.013	Harmful
	Intra-layer PD (6 mm × 6 mm)	0.452	0.045	Harmful
SupraMarginal-L				
	Intra-layer VD (6 mm × 6 mm)	0.48	0.024	Harmful
	Outer VD (6 mm × 6 mm)	0.454	0.034	Harmful
	Completed VD (6 mm × 6 mm)	0.444	0.038	Harmful
Postcentral_R				
	Intra-layer PD (3 mm × 3 mm)	0.489	0.021	Harmful
	Completed PD (3 mm × 3 mm)	0.478	0.024	Harmful

Decreased connections in visual network and its correlation to retinal vascular structures in Healthy control and cognitive impairments.

## Data Availability

In the event that individuals express interest in extending the scope of their data for the purpose of additional validation, the corresponding author is able to provide access to the magnetic resonance imaging (MRI) images to the scientific community.
